# Synthesis, Characterization, and Microwave Absorption Properties of Reduced Graphene Oxide/Strontium Ferrite/Polyaniline Nanocomposites

**DOI:** 10.1186/s11671-016-1340-x

**Published:** 2016-03-12

**Authors:** Juhua Luo, Pan Shen, Wei Yao, Cuifeng Jiang, Jianguang Xu

**Affiliations:** School of Materials Engineering, Yancheng Institute of Technology, Yancheng, 224051 China

**Keywords:** Composite materials, Chemical synthesis, Magnetic measurements, Microwave absorption

## Abstract

Strontium ferrite nanoparticles were prepared by a coprecipitation method, and reduced graphene oxide/strontium ferrite/polyaniline (R-GO/SF/PANI) ternary nanocomposites were prepared by in situ polymerization method. The morphology, structure, and magnetic properties of the ternary nanocomposites were investigated by X-ray powder diffraction (XRD), Fourier transform infrared spectroscopy (FT-IR), TEM, Raman, and VSM. The microwave-absorbing properties of the composites were measured by a vector network analyzer. The XRD patterns show the single phase of strontium hexaferrite without other intermediate phases. TEM photographs reveal that strontium ferrite nanoparticles are uniformly dispersed on the surfaces of R-GO sheets. The R-GO/SF/PANI nanocomposite exhibited the best absorption property with the optimum matching thickness of 1.5 mm in the frequency of 2–18 GHz. The value of the maximum RL was −45.00 dB at 16.08 GHz with the 5.48-GHz bandwidth. The excellent absorption properties of R-GO/SF/PANI nanocomposites indicated their great potential as microwave-absorbing materials.

## Background

In recent years, the rapid growth of electronic equipment has put humans in increasing contact with each other, but it also results in serious electromagnetic pollution in civil and military applications, which is known as electromagnetic interference (EMI). EMI not only affects the functioning of electronic equipment but also causes harmful effects to the health of humans [1, 2]. To solve the EMI problem, the development of microwave-absorbing materials with strong absorption over a broad frequency range is required urgently. Over the past decades, ferrite absorbers [3–5], ferroelectric materials [6, 7], conductive polymers [8–10], and composite materials [11, 12] have been researched, but the traditional microwave-absorbing materials cannot meet all of the requirements such as strong absorption, wide range of absorption frequency, thin thickness, and light weight at the same time [13]. The previous reports have demonstrated that incorporating magnetic particles and dielectric material achieves great enhancement in microwave absorption properties [14–18].

Saini and coworkers reported a composite of polyaniline-coated M-Ba-ferrite powders, the composite obtained improving microwave absorption properties due to the interaction and interfacial polarization between polyaniline and M-Ba-ferrite [19]. Yuan and coworkers synthesized polyaniline (PANI)/SrFe_12_O_19_ composites by in situ polymerization, and their results indicated that the conductivity of PANI on SrFe_12_O_19_ dramatically affected the microwave properties [20]. However, the percolation threshold of PANI is high because of low compatibility and low aspect ratio of the conducting polymer. Moreover, the dispersion of magnetic particle in the composites is also a bottleneck problem. These drawbacks of the composites restrict their application in the microwave absorption field. As the thinnest and most lightweight material in the carbon world, reduced graphene oxide (R-GO), which has extremely a high specific surface area and unique two-dimensional structure, may be the best candidate of electromagnetic wave-absorbing materials [21, 22]. Adding R-GO into the PANI/magnetic particles composites might be an efficient way to overcome these disadvantages due to its high specific surface area and excellent electronic conductivity. The novel ternary composites consisting of R-GO, different magnetic particles, and PANI have been seldom reported so far. Furthermore, the contributions of impedance match and interfacial effects to enhance the microwave absorption performance were also explored.

Therefore, in this paper, we attempted to synthesize the novel kind of R-GO/SF/PANI nanocomposites. The morphology and structure of the ternary nanocomposites were investigated. Furthermore, the contributions of impedance match and synergistic effects to enhance the microwave absorption properties were explored in detail.

## Methods

Graphene oxide (GO) was prepared from purified natural graphite by using modified Hummers method as reported elsewhere [23]. In brief, this method consists of stirring graphite powder in a solution with strong oxidizing agents such as potassium permanganate (KMnO_4_, 99.5 %) and concentrated sulfuric acid (H_2_SO_4_, 98 %). After oxidation, the precipitate was collected by centrifugation and then was washed with distilled water and ethanol to remove metal ions and excess acid until the pH = 7. Then the precipitate was dried at 60 °C for 12 h in vacuum. The product was exfoliated under sonication for about 2 h to ensure most graphite oxide was exfoliated to single-layer graphene oxide.

Desired amounts of strontium nitrate (Sr(NO_3_)_2_) and iron nitrate (Fe(NO_3_)_3_), with Fe^3+^/Sr^2+^ molar ratio of 10.5, were mixed to yield a clear aqueous solution. The mixed solution was added dropwise to the aqueous solution of 10 % excess of sodium hydroxide (NaOH) and sodium carbonate (Na_2_CO_3_) using vigorous stirring to obtain the precipitate. The pH and temperature of the solution during coprecipitation were kept as 13 and 37 °C, respectively. After precipitation, the precipitate was obtained by filtrating and washed with distilled water and ethanol. Then the precipitate was dried at 60 °C in vacuum for 24 h to obtain the precursor. The precursor was calcined at 500 °C for 10 h and then calcined at 900 °C for 2 h to obtain strontium ferrite (SF) nanoparticles.

Cetyltrimethylammonium bromide (CTAB) was added into the GO solution with constant stirring to form a homogeneous dispersion (m(CTAB):m(GO) = 0.8 %). As-prepared SF nanoparticles (m(GO)/m(SF) = 2, 5, 7, 10 %) were added into the above dispersion with vigorous stirring for 12 h. Then hydrazine hydrate (m (hydrazine hydrate)/m(GO) = 0.7) was added into the suspension. Then the suspension was heated at 95 °C with stirring for 1 h. The suspension was centrifugally washed using distilled water and ethanol. The resulting product was dried at 60 °C in vacuum for 12 h to obtain R-GO/SF nanocomposites.

One milliliter aniline monomer and R-GO/SF nanocomposites (m(AN):m(R-GO/SF) = 2:1) were added in 35 ml hydrochloric acid solution (0.1 mol L^−1^). Then the mixture solution was dispersed by ultrasonic wave for 30 min. Ammonium persulfate (2.49 g) was dissolved in 15 ml hydrochloric acid solution (0.1 mol L^−1^). The ammonium persulfate solution was then slowly added dropwise to the above mixture solution with stirring for 12 h. The reaction mixture was centrifugally washed using distilled water and ethanol. The resulting product was dried at 60 °C in vacuum for 24 h to obtain R-GO/SF/PANI nanocomposites. The samples with different weight ratios of 2, 5, 7, and 10 % of GO and SF were denoted as R-GO/SF/PANI-1, R-GO/SF/PANI-2, R-GO/SF/PANI-3, and R-GO/SF/PANI-4, respectively.

### Characterization

The resulting powder was characterized by X-ray powder diffraction (XRD) using a diffractometer (RIGAKU, model D/max) with CuK_α_ radiation of wavelength λ = 1.5418 Å. Its morphology was studied with a field emission scanning electron microscope (JEOL, model JSM-7001F) and a transmission electron microscope (JEOL, model JEM 2001). Fourier transform infrared spectroscopy (FT-IR) for the prepared samples were carried out using the infrared spectrophotometer (NICOLET, model NEXUS 670) in the range from 4000 to 400 cm^−1^. Raman spectra were measured using a laser Raman spectrometer (Thermo Fisher, model DXR) at a 663-nm wavelength incident laser light. Magnetization measurements were taken at room temperature (293 K) using a vibrating sample magnetometer (LDJ, model 9600–1). The complex permittivity (${\varepsilon}_{\mathit{\mathsf{r}}}={\varepsilon}^{\hbox{'}}-\mathit{\mathsf{j}}{\varepsilon}^{\hbox{'}\hbox{'}}$) and permeability (${\mu}_{\mathit{\mathsf{r}}}={\mu}^{\hbox{'}}-\mathit{\mathsf{j}}{\mu}^{\hbox{'}\hbox{'}}$) of the samples were measured by a microwave vector network analyzer (AGILENT, model N5244A) in the frequency range 2–18 GHz by using coaxial reflection/transmission technique. The samples for vector network analyzer were pressed to be toroidal samples with OD 7 mm, ID 3.04 mm, and height at 3 mm according to the mass ration 1:1 of paraffin and R-GO/SF/PANI nanocomposites.

## Results and Discussion

### Structure and Morphology Analysis

**Fig. 1:**
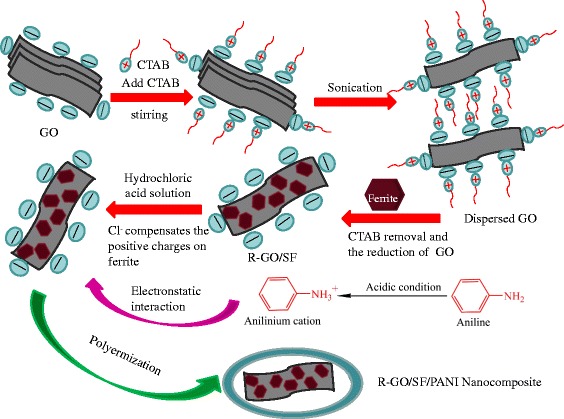
Schematic representation of the preparation of the R-GO/SF/PANI nanocomposites

The preparation of the R-GO/SF/PANI nanocomposites is illustrated in Fig. 1. As it is well known that the surface of GO contain many oxygenated functional groups (such as –OH and –COOH) and show electrostatic in solution, we believe that CTAB as a cationic surfactant can play an important role in the dispersion of R-GO in aqueous solution. The interaction of CTAB with R-GO is assumed to be electrostatic in nature, which helps to break the van der Waals forces between the layers of GO [24]. It is known to us that the surface charge of metal oxide is positive below the pH of the point of zero charge (PZC), while it becomes negative above PZC. Since the surface of magnetite has PZC of pH = 6 [25]. The GO solution is weakly acidic; when SrFe_12_O_19_ is dispersed in GO solution, the surface of SrFe_12_O_19_ with positive charge is attracted by the surface of GO with electronegativity and automatic assembly on the surface of GO sheets and forming R-GO/SF nanocomposites. In the preparation process of R-GO/SF/PANI nanocomposites, the co-initiator additive HCl (0.1 mol L^−1^) makes the solution acidic in condition; Cl^−1^ is absorbed and compensates the positive charge on SrFe_12_O_19_ nanoparticles. Finally, the surface of SrFe_12_O_19_ gathering negative charge and the surface of R-GO/SF nanocomposites are electronegative. Aniline monomers are converted to cationic anilinium ions in acidic conditions, which is absorbed on the surface of SrFe_12_O_19_ composites and polymerized to form R-GO/SF/PANI nanocomposites [26]. Besides, Singh demonstrated that the solid state charge transfer between R-GO and PANI is also conducive to form the stable R-GO/SF/PANI nanocomposites [27].

**Fig. 2:**
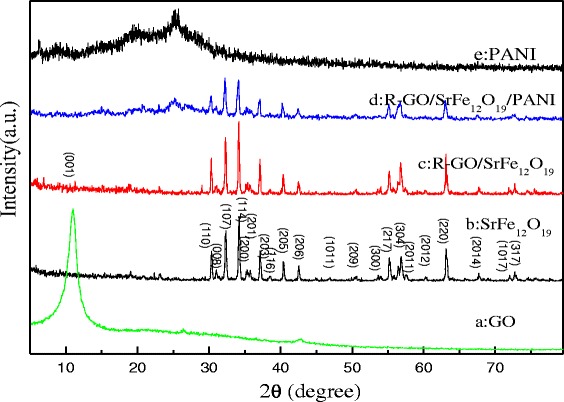
XRD patterns of (*a*) GO, (*b*) SrFe_12_O_19_, (*c*) R-GO/SF nanocomposites, (*d*) R-GO/SF/PANI nanocomposites, and (*e*) PANI

Figure 2 shows the XRD patterns of GO, SrFe_12_O_19_, R-GO/SF nanocomposites, R-GO/SF/PANI nanocomposites, and PANI. As show in Fig. 2, the XRD pattern of the PANI shows amorphous nature in a partially crystalline state with two diffraction peaks at 20.15° and 25.34° [28, 29]. From the XRD curve of GO in Fig. 2(a), it can be seen that the appearance of strong sharp diffraction peak at 2θ = 10.96° is corresponding to the (001) plane of GO [19], which means that natural graphite has been oxidized into GO with regular crystal structure and high-degree oxidation [30]. Figure 2(b) shows the XRD pattern of SrFe_12_O_19_; it can be seen that SrFe_12_O_19_ is M-type ferrite (PDF Card no.33-1340). The series sharp diffraction peaks of SrFe_12_O_19_ at 2θ = 30.2°, 32.1°, 34.1°, 37.0°, 40.3°, and 42.3° are assigned to the (100), (008), (107), (114), (008), (200), and (201) crystal planes, respectively [31, 32]. Figure 2(c) shows the XRD pattern of R-GO/SF nanocomposites in which the characteristic diffraction peaks of SrFe_12_O_19_ can be clearly observed. Compared with the standard diffraction peak characteristic spectral lines of SrFe_12_O_19_, the diffraction peak of R-GO/SF nanocomposites shifts towards lower 2θ and the diffraction peak of GO in the R-GO/SF nanocomposites disappeared. Based on previous reports [33], GO is effectively reduced to R-GO under the effect of reducing agent hydrazine hydrate, and the lamellar dispersion of R-GO in R-GO/SF composites is well. The characteristic diffraction peak of SrFe_12_O_19_ in the R-GO/SF composites shift slightly towards lower 2θ, in which it can be deduced that SrFe_12_O_19_ has been successfully assembled on the surface of R-GO sheets [34, 35]. In Fig. 2(d), not only the characteristic diffraction peaks of R-GO/SF nanocomposites were observed but also the characteristic diffraction peaks of PANI [33], which means the presence of R-GO, SrFe_12_O_19_, and PANI in the R-GO/SF/PANI nanocomposites.

**Fig. 3:**
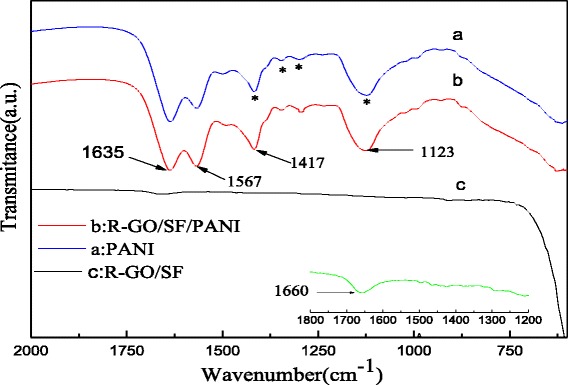
FT-IR spectra of (*a*) PANI, (*b*) R-GO/SF/PANI nanocomposites, and (*c*) R-GO/SF nanocomposites

Figure 3 shows the FT-IR spectra of PANI, R-GO/SF nanocomposites, and R-GO/SF/PANI nanocomposites. The characteristic absorption peak of oxygen-containing functional groups on the GO is not observed in the FT-IR spectrum of R-GO/SF nanocomposites (Fig. 3(c)), indicating that GO is effectively reduced into R-GO. In Fig. 3(c), the characteristic absorption peak at 1660 cm^−1^ is attribute to the C=C skeleton vibration of the sp^2^ hybridized of unoxidized graphite [36]. The FT-IR spectrum of R-GO/SF/PANI nanocomposites is shown in Fig. 3(b). It can be clearly observed the characteristic absorption peak of PANI from the curve of Fig. 3(c). The characteristic peaks at 1635 and 1567 cm^−1^ are attributed to the stretching vibration of quinoid and benzenoid rings on PANI molecular chain, respectively [26]. The characteristic peak at 1417 cm^−1^ is corresponding to the stretching mode of N–Q–N where Q represents the benzenoid ring [37]. The characteristic peak at 1344 and 1295 cm^−1^ corresponds to N–H bending and asymmetric C–N stretching mode for benzenoid ring, respectively [38]. The peak at 1123 cm^−1^ is attributed to aromatic C–H inplane bending mode [39]. Compared with the FT-IR spectrum of PANI (Fig. 3(a)), the characteristic peaks in the FT-IR spectrum of R-GO/SF/PANI nanocomposites are slightly red shifted and observe the C=C skeleton vibration on R-GO. The reasons of the FT-IR spectrum of R-GO/SF/PANI nanocomposite red shift may have three points: (1) the π molecular orbital of PANI overlaps the empty *d*-orbital of Fe^3+^ in strontium ferrite to form the σ-bond where metal ions play a role of the electron pair acceptor; (2) the π* molecular orbital of PANI overlaps the empty *d*-orbital of Fe^3+^ to form the π-bond, in which the Fe^3+^ is the electron pair donor; and (3) the presence of solid state charge transfer in R-GO and PANI, R-GO as the cation acceptor, the surface of sheets has electronegativity, but the existence of emeraldine salt form of PANI as cation having electropositivity. Therefore, the electron transfer balance between the PANI molecular chain and surface of R-GO easily forms electron transfer complex [27].

**Fig. 4:**
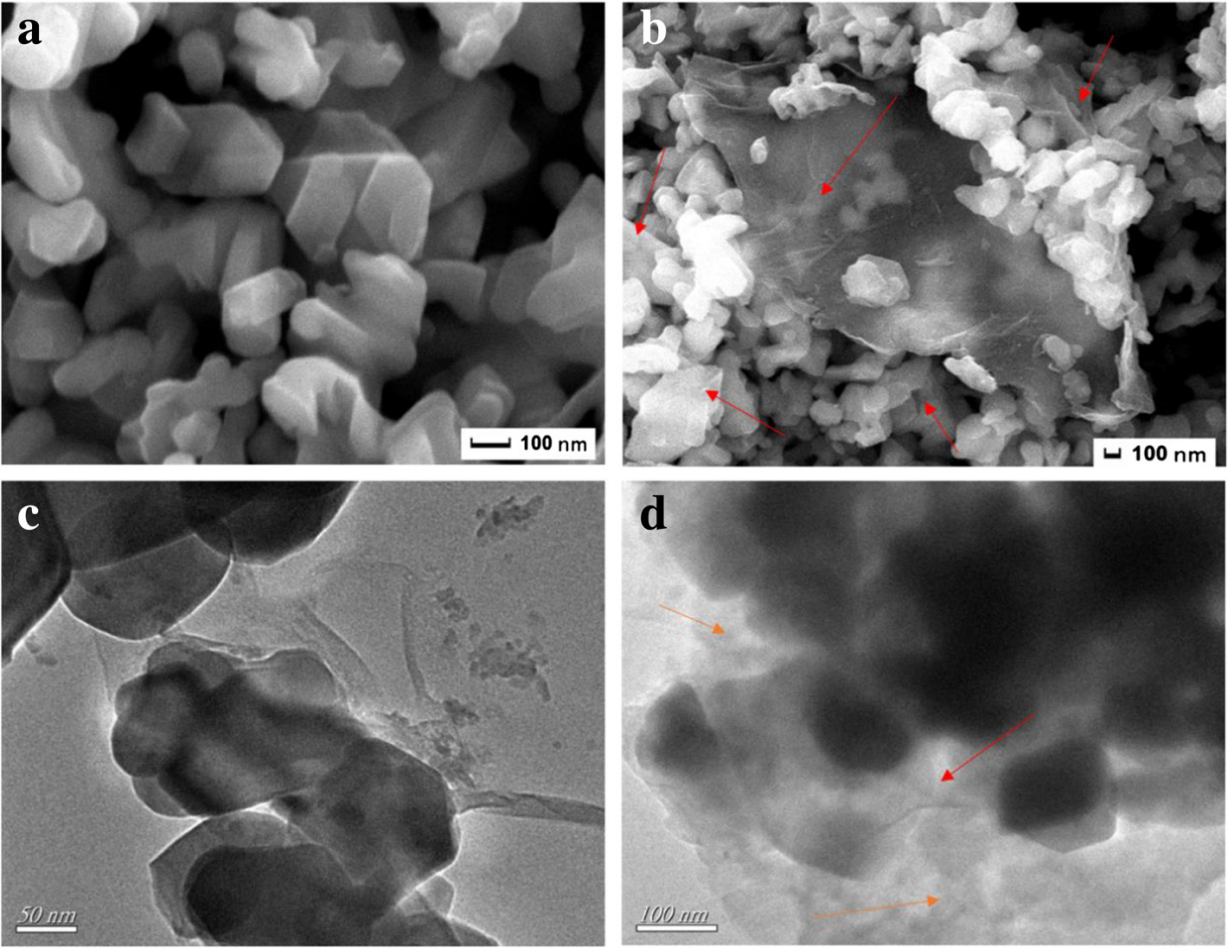
FESEM images of **a** SrFe_12_O_19_ nanoparticles, **b** R-GO/SF nanocomposites and TEM images of **c** R-GO/SF nanocomposites and **d** R-GO/SF/PANI nanocomposites

The FESEM and TEM images of SrFe_12_O_19_ nanoparticles, R-GO/SF nanocomposites, and R-GO/SF/PANI ternary nanocomposites are shown in Fig. 4. Figure 4a shows the micromorphology of SrFe_12_O_19_ nanoparticles; the hexagonal structure of ferrite can be clearly observed and aggregate due to the magnetic dipole interaction between ferrite particles [38]. The average particle size induced from the TEM micrograph was in the range 50–100 nm. The selected area electron diffraction (SAED) pattern further indicates that the ferrite particle is highly crystalline with M-type ferrite. The FESEM image of R-GO/SF nanocomposites shown in Fig. 4b clearly displays that R-GO sheets are transparent and wrinkled. It also can be seen that SrFe_12_O_19_ nanoparticles are uniformly dispersed on the surface of R-GO sheets. In order to observe the micromorphology of SrFe_12_O_19_ nanoparticles, R-GO/SF nanocomposites, and R-GO/SF/PANI ternary nanocomposites, the FESEM and TEM images are shown in Fig. 4. In the TEM image of R-GO/SF/PANI nanocomposites shown in Fig. 4d, it can be observed that the surface of R-GO/SF nanocomposites is uniformly coated by PANI molecular chains.

**Fig. 5:**
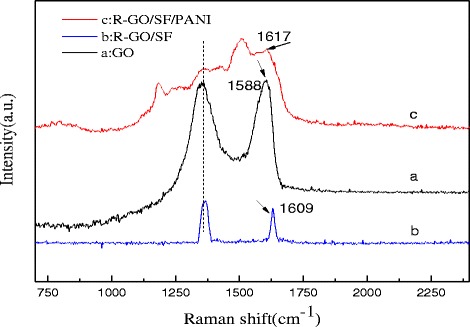
Raman spectra of (*a*) GO, (*b*) R-GO/SF nanocomposites, and (*c*) R-GO/SF/PANI nanocomposites

Figure 5 shows the Raman spectra of GO, R-GO/SF nanocomposites, and R-GO/SF/PANI nanocomposites. The characteristic features in the Raman spectrum of GO are the so-called D band, which locate at around 1310 cm^−1^, corresponding to the breathing mode of k-point phonons of *A*_1g_, and the G band at 1598 cm^−1^ is attributed to the tangential stretching mode of the *E*_2g_ phonons of sp^2^ atoms [40]. The characteristic D and G peaks of R-GO being present in the Raman spectra of R-GO/SF nanocomposites and R-GO/SF/PANI nanocomposites and the intensity of peaks decreased with an increase in R-GO content. In Fig. 5(a, b, c), it can be observed that the G band of R-GO/SF nanocomposites and R-GO/SF/PANI nanocomposites (clarity declined due to overlap of the characteristic peaks of PANI) experienced a shift of about 9 cm^−1^ and 8 cm^−1^ compared to R-GO, respectively, indicating the presence of charge transfer among R-GO, SF, and PANI. This is consistent with the previous reports [28, 41].

### Magnetic Properties

**Fig. 6:**
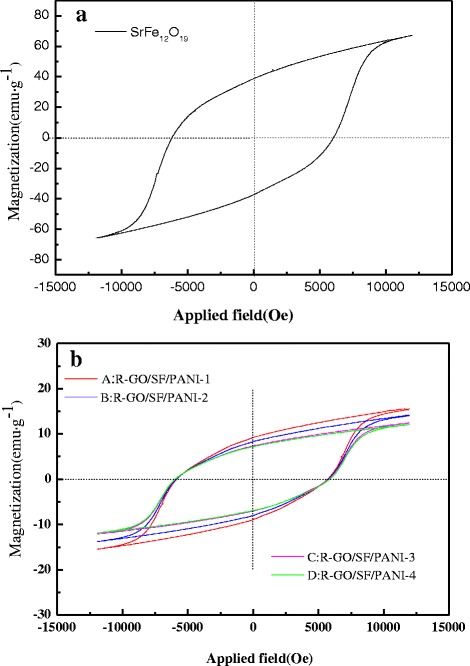
The magnetization hysteresis loops of **a** SrFe_12_O_19_ nanoparticles and **b** R-GO/SF/PANI nanocomposites with different rations

Figure 6a shows hysteresis loop of SrFe_12_O_19_ nanoparticles. SrFe_12_O_19_ magnetic nanoparticles are the permanent magnet and show the ferromagnetic, the hysteresis loop with high saturation magnetization (M_s_) and the value is 67.15 emu g^−1^, which is close to the theoretical value of the saturation magnetization of M-type strontium ferrite (74.3 emu g^−1^). This fully shows that the preparation of SrFe_12_O_19_ nanoparticles are pure phase and no nonmagnetic impurity phase α-Fe_2_O_3_ exist in the product; it is consistent with the XRD analysis [26]. The coercivity (H_c_) and remanence (M_r_) of SrFe_12_O_19_ are 6022 Oe and 39.09 emu · g^−1^, respectively. The squareness ratio (S) was calculated by the Stoner-Wohlfarth model, *S* = *M*_r_/*M*_s_ = 0.58, the value of *S* is slightly higher than the previous work [42, 43], which means SrFe_12_O_19_ has more excellent magnetic properties. Figure 6b shows hysteresis loops of SrFe_12_O_19_ nanocomposites, and the magnetic parameters are shown in Table 1. It can be observed that the saturation and remanent magnetization are dropped dramatically with an increase in the nonmagnetic R-GO and PANI and decreased with increase in the R-GO content of the composites; the coercivity of R-GO/SF/PANI nanocomposites declined slightly compared with pure SrFe_12_O_19_ magnetic nanoparticles; it also decreased with the increase in the R-GO content of the composites and gradually showed the magnetic characteristic of paramagnetic; the phenomenon is similar with a previous work [41].

**Table 1:** Magnetic parameters of R-GO/SF/PANI nanocomposites

Sample no.	M_s_ (emu g^−1^)	H_c_(Oe)	M_r_ (emu ^−1^)
R-GO/SF/PANI-1	15.75	5854	9.28
R-GO/SF/PANI-2	14.23	5756	8.36
R-GO/SF/PANI-3	12.46	5720	7.40
R-GO/SF/PANI-4	12.16	5692	7.23

### Microwave Absorption Properties

**Fig. 7:**
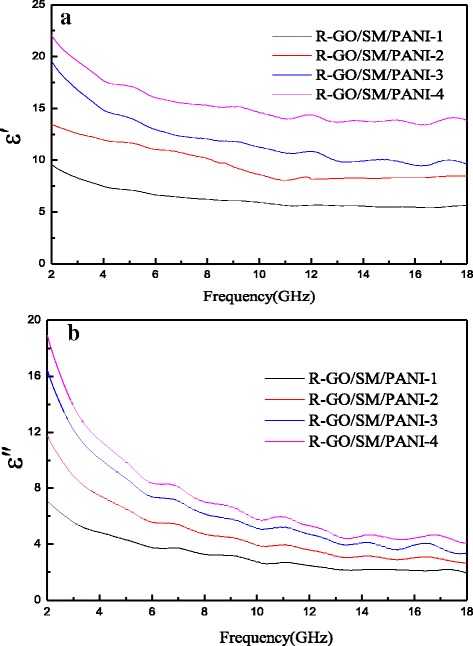
Behavior of **a** real and **b** imaginary part of the permittivity of R-GO/SF/PANI-1, R-GO/SF/PANI-2, R-GO/SF/PANI-3, and R-GO/SF/PANI-4 composites as a function of frequency over 2–18 GHz

The complex permittivity real part (ε′) and imaginary part (ε″) of R-GO/SF/PANI nanocomposites with different rations are shown in Fig. 7a, b, respectively. In Fig. 7, it can be seen that the ε′ and ε″ values of nanocomposites with different rations decreased with an increase in frequency and increased with an increase in the R-GO content. As shown in Fig. 7a, it can be seen that the ε′ values of R-GO/SF/PANI-1, R-GO/SF/PANI-2, R-GO/SF/PANI-3, and R-GO/SF/PANI-4 decreased gradually from 9.58 to 5.61, 13.58 to 9.61, 19.56 to 9.65, and 22.06 to 13.87, respectively. In Fig. 7b, it can be observed that the ε″ values of R-GO/SF/PANI-1, R-GO/SF/PANI-2, R-GO/SF/PANI-3, and R-GO/SF/PANI-4 decrease gradually from 7.09 to 1.96, 11.10 to 5.96, 15.99 to 3.39, and 18.94 to 4.08, respectively. The dielectric loss of the composites could be explicated by the Debye theory [17, 21, 44, 45]. The ε″ is known as

1 $$ {\varepsilon}^{{\prime\prime} }=\left({\varepsilon}_{\mathit{\mathsf{s}}}-{\varepsilon}_{\infty}\right)\times \omega \tau /\left(\mathsf{1}+{\omega}^{\mathsf{2}}{\tau}^{\mathsf{2}}\right)+\sigma /\omega {\varepsilon}_{\mathsf{0}} $$

where σ is the dc conductive of the composites. Due to the recovery of the electric conductivity after chemical reduction and thinning, the increased polarization caused by the abundant surface functional groups enhances the dielectric loss. On the contray, R-GO constract more conductive paths in the composites for electron transport, which maks a significant contribution to dielectric loss [44].

**Fig. 8:**
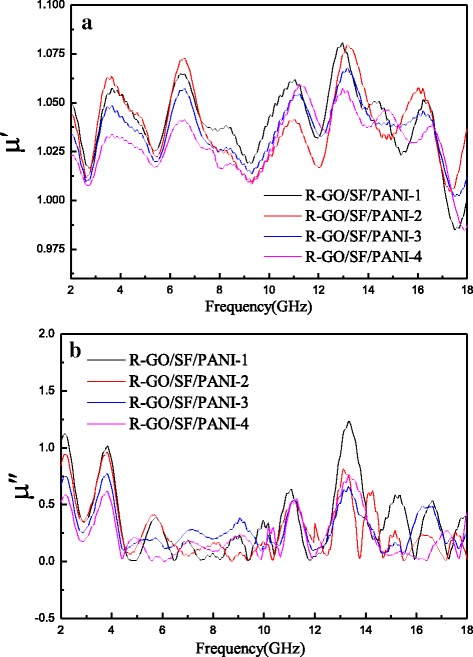
Behavior of **a** real and **b** imaginary part of the permeability of R-GO/SF/PANI-1, R-GO/SF/PANI-2, R-GO/SF/PANI-3, and R-GO/SF/PANI-4 composites as a function of frequency over 2–18 GHz

Figure 8 shows the complex permeability real part (μ′) and imaginary part (μ″) of R-GO/SF/PANI nanocomposites with different rations as a function of frequency over 2–18 GHz. It can be seen that the complex permeability real part (μ′) and imaginary part (μ″) of R-GO/SF/PANI nanocomposites with different rations have the same trend and change in volatility; the values of μ′ change have small amplitude and little influence by the R-GO content and fluctuating in the range of 1–1.05; the values of μ″ fluctuation tendency is clear and fluctuating in the range of 0–1.

Calculation for the microwave absorption of the composites was carried out based on the experimentally determined complex permittivity and permeability. The reflection loss (RL) can be calculated as [17]:

2 $$ \mathit{\mathsf{R}}\mathit{\mathsf{L}}=\mathsf{20} \log \left|\frac{{\mathit{\mathsf{z}}}_{\mathit{\mathsf{in}}}-\mathsf{1}}{{\mathit{\mathsf{z}}}_{\mathit{\mathsf{in}}}+\mathsf{1}}\right| $$

**Fig. 9:**
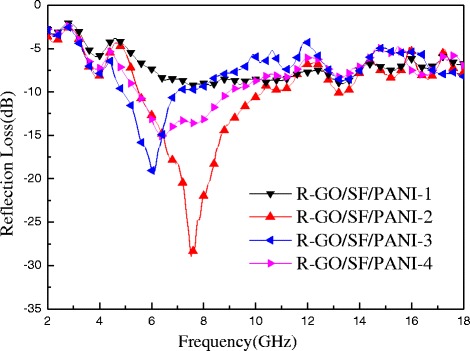
The calculated reflection loss curves of R-GO/SF/PANI nanocomposites with different components

Here, the normalized input impedance Z_in_ of microwave absorption layer is as follow

3 $$ {\mathit{\mathsf{z}}}_{\mathit{\mathsf{in}}}=\sqrt{\frac{\mu_{\mathit{\mathsf{r}}}}{\varepsilon_{\mathit{\mathsf{r}}}}} \tanh \left[\mathit{\mathsf{j}}\frac{\mathsf{2}\pi \mathit{\mathsf{f}}\mathit{\mathsf{d}}}{\mathit{\mathsf{c}}}\sqrt{\mu_{\mathit{\mathsf{r}}}{\varepsilon}_{\mathit{\mathsf{r}}}}\right] $$

where *f* is the frequency of incident electromagnetic wave, *d* is the absorber thickness, *c* is the velocity of light, and *ε*_r_ and *μ*_r_ are the complex permittivity and permeability of the composites medium, respectively. Figure 9 shows reflection loss curves of R-GO/SF/PANI nanocomposites with different components at matching thickness of 3 mm. As shown in Fig. 9, it can be observed that R-GO/SF/PANI-2 possesses the best absorbing properties. The maximum RL of R-GO/SF/PANI-2 nanocomposite is −28.95 dB at 7.52 GHz, and the bandwidth of below −10 dB is 4.74 GHz. The absorbing property of R-GO/SF/PANI-1 nanocomposite is the worst, and the maximum RL value is only −8.56 dB. The values of the maximum RL are −19.04 dB at 6.04 GHz with the 2.04 GHz bandwidth and −15.06 dB at 6.36 GHz with the 3.52-GHz bandwidth for the R-GO/SF/PANI-3 and R-GO/SF/PANI-4, respectively.

**Fig. 10:**
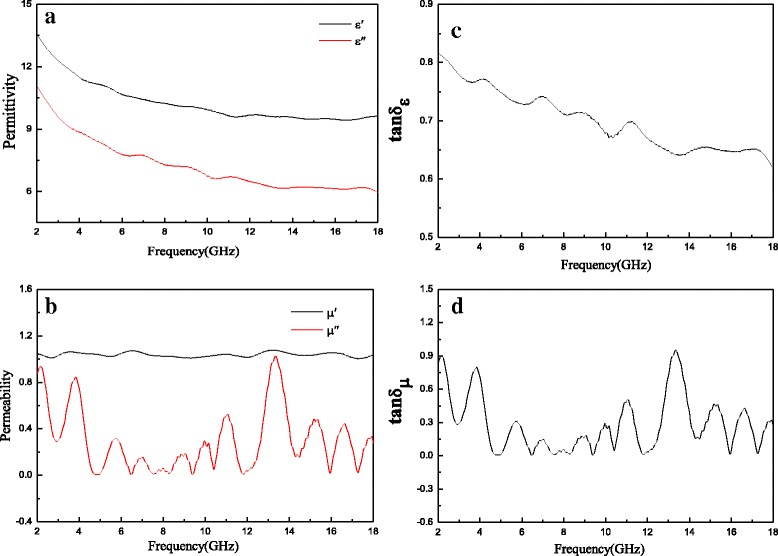
The electromagnetic parameters of **a** the real and imaginary part of complex permittivity, **b** the real and imaginary part of complex permeability, **c** dielectric loss tangent, and **d** magnetic loss tangent of R-GO/SF/PANI-2 nanocomposite

The real and imaginary part of complex permittivity and dielectric loss tangent of R-GO/SF/PANI-2 nanocomposite are shown in Fig. 10a, c, respectively. As shown in Fig. 10a, it can be observed that the complex permittivity real part (ε′) is exponential decline in the frequency range of 2–18 GHz and the values decrease from 13.58 to 9.61. The imaginary part also shows the same variation and the values decrease from 11.10 to 5.96. The reasons of the complex permittivity real and imaginary part varying with frequency may be attributed to the following two points. (1) With an increase in the frequency of external reverse electric field, the induction charge phase of R-GO/SF/PANI-2 nanocomposite behind the external electric field and results in electromagnetic oscillation [46], the values of ε′ and ε″ decrease with an increase frequency. (2) The dielectric performance of the composites is influenced by the space charge polarization. The space charge polarization is associated with the heterogeneity and present at the interface among the components of the composites. The difference in dielectric constants among the components of R-GO/SF/PANI-2 nanocomposite is responsible for the generation of space charge polarization. The space charge polarization decreases with an increase in the frequency, which results in the values of ε′ and ε″ decrease with an increase in frequency [27]. The dielectric loss tangent (tanδε) also shows the same trend and the values decrease from 0.82 to 0.62.

The real and imaginary part of complex permeability curves of R-GO/SF/PANI-2 nanocomposite in the frequency range of 2–12 GHz were measured and shown in Fig. 10b. As shown in Fig. 10b, it can be seen that the real part (μ′) almost is a constant in whole range, and the values of μ′ are floating around 1 and the floating modest. The imaginary part (μ″) in whole range of 2–12 GHz shows the obvious fluctuation and the values are fluctuating between 1.03 and 0.01. In Fig. 10d, the magnetic loss tangent also shows the similar trend. Generally, the magnetic loss of magnetic materials is originated from hysteresis loss, domain-wall resonance, eddy current effect, and natural resonance [47]. The hysteresis loss of strontium ferrite in R-GO/SF/PANI-2 nanocomposite can be ignored in the frequency range of 2–18 GHz, the domain-wall resonance of strontium ferrite usually occurs in lower frequency than the GHz range [48, 49]. Therefore, Eddy current effect and natural resonance are the main magnetic loss of R-GO/SF/PANI-2 nanocomposite.

**Fig. 11:**
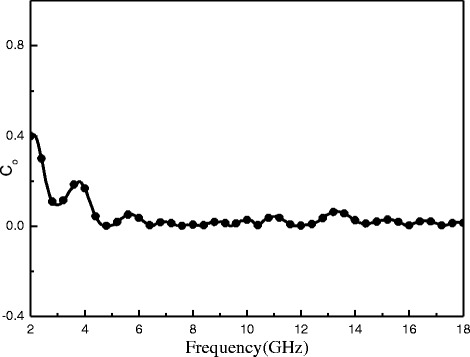
The Co-f values of R-GO/SF/PANI-2 nanocomposite

The magnetic loss caused due to Eddy current effect can be calculated as [50] ${\mathit{\mathsf{C}}}_{\mathsf{0}}={\mu}^{\hbox{'}\hbox{'}}{\left({\mu}^{\hbox{'}}\right)}^{-\mathsf{2}}{\mathit{\mathsf{f}}}^{-\mathsf{1}}$. The skin-effect criterion shows that the values of C_0_ should be constant when the frequency varies, if the magnetic loss results from Eddy current effect. Figure 11 shows the curve of C_0_ with frequency for R-GO/SF/PANI-2 nanocomposite. As shown in Fig. 11, it can be observed that C_0_ significantly decreased at the frequency range of 2–6 GHz. However, C_o_ is approximated constant in the frequency range of 6–18 GHz. Therefore, the magnetic losses of 2–6 and 6–18 GHz are mainly caused by natural resonance and eddy current effect, respectively. Therefore, the resonance peak observed at 3.84 GHz is caused by natural resonance [50]. The other resonance peaks observed at 6–18 GHz are caused by Eddy current effect.

**Fig. 12:**
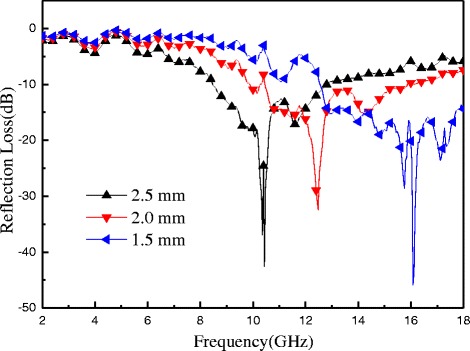
The calculated reflection loss curves of R-GO/SF/PANI-2 nanocomposite

In order to study the microwave absorption performance in-depth, Fig. 12 shows the reflection losses of R-GO/SF/PANI-2 nanocomposite with different matching thickness of 1.5, 2.0, and 2.5 mm. As shown in Fig. 12, R-GO/SF/PANI-2 nanocomposite has the best microwave-absorbing properties and the maximum RL value is −45.00 dB at 16.08 GHz, the reflection loss of R-GO/SF/PANI-2 nanocomposite below −10 dB at 12.52–18.00 GHz with the bandwidth of 5.48 GHz. When the matching thickness is up to 2.0 mm, the bandwidth with the reflection loss of R-GO/SF/PANI-2 nanocomposite below −10 dB is up to 5.84 GHz at 9.88–10.32 GHz and 10.48–15.88 GHz, but the maximum reflection loss is −32.42 dB at 12.48 GHz. When the matching thickness is 2.5 mm, the maximum reflection loss is shifted to 10.44 GHz and the value is 42.61 dB, and the absorption bandwidth with the reflection loss below −10 dB is 4.28 GHz (from 8.48 to 12.76 GHz). According to the following formula [51]: ${\mathit{\mathsf{f}}}_{\mathit{\mathsf{m}}}=\frac{\mathit{\mathsf{c}}}{\mathsf{2}\pi {\mu}^{\hbox{'}\hbox{'}}\mathit{\mathsf{d}}}$, where *f*_m_ is the frequency of the maximum reflection loss peak and *d* is the matching thickness, it is obvious that the matching thickness for absorbing properties of nanocomposites has a regulatory role and reflection loss peaks move to low frequency with the increasing matching thickness.

The absorbing property of pure SrFe_12_O_19_ nanoparticles is poor, the maximum reflection loss is only −6.50 dB, and bandwidth under −10 dB is nearly 0 GHz [52]. The absorbing properties of PANI/SrFe_12_O_19_ nanocomposites were improved when SrFe_12_O_19_ was packed by PANI, the maximum reflection loss is up to −11.73 dB, and bandwidth under −10 dB is 0.6 GHz [53]. The absorbing properties of PANI/SrFe_12_O_19_ are far less than that of R-GO/SF/PANI. Synergistic effects of R-GO, SF, and PANI play an important role for enhancing the absorbing properties of R-GO/SF/PANI nanocomposites. Firstly, the 2D-structure R-GO with large specific surface area can form a complete conductive network, which can improve the dielectric loss. Moreover, the absence of structure and residual functional groups on the surface of R-GO can improve the matching characteristics [54]. Secondly, the SF nanoparticles absorbed on the surface of R-GO have high dispersion, which can improve magnetic loss [55]. When the incident electromagnetic wave hits the R-GO/SF/PANI nanocomposites, oscillating current was formed due to movement of carrier of R-GO; dielectric relaxation and dielectric polarization are triggered because of interface charge of R-GO. The SF nanoparticles absorbed on the surface of R-GO can also be used as multi-pole polarization center, strengthening the electronic polarization of nanocomposites and regulating the incident electromagnetic wave, which is conducive to strong absorption of electromagnetic wave [56]. Finally, the presence of PANI coating layer enhances the Debye dipole relaxation of R-GO, the conjugated electron clouds of PANI molecular chains are transferred to R-GO by electronic polarization to form electron tunneling between PANI and R-GO, which has the tunnel effect and enhances the absorption of R-GO/SF/PANI nanocomposites for electromagnetic wave; this is related to the previous work [57, 58]. Moreover, the absorbing materials not only require a single high dielectric loss and magnetic loss but also have excellent matching characteristic, namely dielectric loss tangent of the materials is close or equal to the magnetic loss tangent. As shown in Fig. 10c, d, R-GO/SF/PANI nanocomposites have excellent matching characteristics, which make a great contribution to improve microwave-absorbing properties.

## Conclusions

Strontium ferrite nanoparticles were well distributed and firmly anchored onto the surface of the R-GO sheets by use CTAB as surfactant, the R-GO/SF/PANI ternary nanocomposites were successfully prepared by an in situ polymerization. The synergistic effect among R-GO, strontium ferrite, and PANI had great influence on enhance microwave absorption properties of R-GO/SF/PANI ternary nanocomposites. The R-GO/SF/PANI-2 nanocomposite possessed the best absorption property. The value of the maximum RL was up to −45.00 dB at 16.08 GHz with a matching thickness of 1.5 mm and the absorption bandwidth with the RL below −10 dB reached 5.8 GHz which covered the whole Ku band. Hence, the R-GO/SF/PANI ternary nanocomposites are promising as the applications of potential microwave absorber materials.
